# Deep brain stimulation for treatment-resistant major depressive disorder: a comparison of two targets and long-term follow-up

**DOI:** 10.1038/tp.2017.66

**Published:** 2017-10-31

**Authors:** S Raymaekers, L Luyten, C Bervoets, L Gabriëls, B Nuttin

**Affiliations:** 1 KU Leuven Research Group Psychiatry, Leuven, Belgium; 2 Z.ORG UPC KU Leuven Campus Gasthuisberg, Leuven, Belgium; 3 KU Leuven Research Group Experimental Neurosurgery and Neuroanatomy, Leuven, Belgium; 4 KU Leuven Research Group Psychology of Learning and Experimental Psychopathology, Leuven, Belgium; 5 UZ Leuven, Department of Neurosurgery, Leuven, Belgium

## Abstract

We previously found that electrical stimulation in the anterior limb of the internal capsule/bed nucleus of the stria terminalis (IC/BST) alleviates depressive symptoms in severe treatment-resistant obsessive-compulsive disorder (OCD) patients. Here we tested the hypothesis that electrical stimulation in either IC/BST or in the inferior thalamic peduncle (ITP) effectively reduces depressive symptoms in treatment-resistant major depressive disorder (TRD). In a double-blind crossover design, the effects of electrical stimulation at both targets were compared in TRD patients. The 17-item Hamilton Depression Rating scale (HAM-D) was the primary outcome measure. During the first crossover, patients received IC/BST stimulation versus no stimulation in random order (2 × 1 weeks). During the second crossover (3 × 2 months), patients received IC/BST versus ITP versus no stimulation. Patients and evaluators were blinded for stimulation conditions. All patients (*n*=7) were followed up for at least 3 years (3–8 years) after implantation. Six patients completed the first crossover and five patients completed the second. During the first crossover, mean (s.d.) HAM-D scores were 21.5 (2.7) for no stimulation and 11.5 (8.8) for IC/BST stimulation. During the second crossover, HAM-D scores were 15.4 (7.5) for no stimulation, 7.6 (3.8) for IC/BST stimulation and 11.2 (7.5) for ITP stimulation. The final sample size was too small to statistically analyze this second crossover. At last follow-up, only one patient preferred ITP over IC/BST stimulation. Two patients, with a history of suicide attempts before implantation, committed suicide during the follow-up phases of this study. Our data indicate that, in the long term, both ITP and IC/BST stimulation may alleviate depressive symptoms in patients suffering from TRD.

## Introduction

Major depressive disorder (MDD) is defined as the presence of depressed mood or anhedonia for more than 2 weeks, accompanied by additional symptoms (for example, suicidal thoughts, sleep disturbances and so on). MDD is a burdensome disorder with a substantial and increasing impact on society,^1, 2^ and a lifetime prevalence of up to 17%,^3^ with recent findings suggesting an even higher prevalence.^4^ Treatment strategies include pharmacotherapy,^5^ psychotherapy,^6^ repetitive transcranial magnetic stimulation (rTMS)^7^ and electroconvulsive therapy (ECT).^8^ Up to 30% of patients with MDD however do not adequately respond to treatment. In these treatment-resistant MDD (TRD) patients, the disorder often takes a chronic, disabling course.^9, 10^

Functional imaging studies have resulted in a better understanding of the brain regions involved in the pathology of MDD.^11^ This in turn has led to a search for treatment options targeting this dysfunctional neurocircuitry. Several potential targets for deep brain stimulation (DBS) have been investigated: the subcallosal cingulate (SCC, Cg 25),^12, 13, 14, 15, 16^ nucleus accumbens (NAc),^17^ ventral capsule/ventral striatum (VC/VS),^18, 19^ and supero-lateral medial forebrain bundle (MFB)^20^ might all be valuable in the treatment of TRD.

Since 1998, we have been investigating DBS in the anterior limb of the internal capsule and the bed nucleus of the stria terminalis (IC/BST), for obsessive-compulsive disorder (OCD).^21, 22, 23, 24^ A sustained, and often immediate, reduction in obsessions, compulsions, anxiety and depressive mood was demonstrated in these OCD patients, particularly when the active stimulation contacts were situated in or near the BST.^22^ The BST is located in the immediate vicinity of the VC/VS and NAc region that has been used in other DBS trials for psychiatric disorders, but meticulous neuroanatomical analyses have indicated that the stimulated region in our patients does not or only partially overlaps with the target in VC/VS studies that provided details about their exact stimulation target.^25^ The BST is part of the limbic system^26, 27^ and, for the present study, this target was mainly chosen because of the beneficial effects on comorbid depressive symptoms in our OCD patients.^24^ Moreover, a strong theoretical argument can be made for the BST as a target for treating TRD, as it has projections to many of the above-mentioned structures (MFB, NAc)^28^ and might function as a relay center in the processing of reward, stress and anxiety.^29^ The potential usefulness of this target is further underlined by the correlation of local field potential power in this area and symptom severity in TRD patients.^30^

A potential disadvantage of DBS at IC/BST is the rather high charge density compared to typical stimulation parameters used for DBS in Parkinson’s disease,^31^ necessitating frequent replacements of the neurostimulators or the use of rechargeable batteries.^22^ To address this issue, we further explored the inferior thalamic peduncle (ITP) as a DBS target. Bilateral ITP stimulation was reported to induce beneficial effects in one TRD patient.^32^ ITP connects the dorsomedial thalamus with the orbitofrontal cortex, which both show hypermetabolism in depressed patients.^33^ ITP is a small structure and might require lower charge densities to be stimulated effectively (for a more detailed description of ITP as a target in MDD patients, we refer to Velasco *et al.*^33^)

Because of the limited experience with ITP stimulation in TRD patients, we opted to directly compare IC/BST and ITP stimulation. We hypothesized that electrical stimulation at both targets would be equally safe and effective, but that ITP stimulation would require lower charge densities, implying longer battery life. To our knowledge, until now, there are no studies comparing different psychiatric DBS targets in the same patient.

## Materials and methods

### Eligibility criteria for participants

Patients were initially screened by a comprehensive review of their psychiatric history, obtained by interviewing the patient, family and treating psychiatrist and/or psychologist, as well as by examining all the available records of previous psychiatric treatment. Inclusion criteria ensured the severity, treatment-refractoriness and incapacitating nature of MDD.

Patients were considered candidates for this trial if they were 18–65 years old, had MDD, unipolar type, diagnosed by the Structured Clinical Interview for DSM-IV (SCID-IV)^34^ and judged to be of disabling severity, with a 17-item Hamilton Depression Rating scale (HAM-D) score of at least 19 and Global Assessment of Function (GAF) score of 45 or less. MDD had to be recurrent (>4 episodes) or chronic (episode duration >2 years). Other requirements were a minimum of 5 years since the onset of the first depressive episode, with documented major impairment in functioning or potentially severe medical outcomes (repeated hospitalizations, serious suicidal ideation or a history of previous suicide attempts or other self-injurious behavior). Treatment history was required to prove failure in response to adequate trials of pharmacotherapy and ECT. For more detailed inclusion and exclusion criteria, see Supplementary Information. Medication was tapered off to a bearable minimum and maintained on a stable regimen throughout the first year of DBS.

Patients were recruited from Belgium and The Netherlands, and were screened and followed up both at the psychiatric department of the University Hospital of Antwerp (until June 2007) and the University Hospitals of Leuven (from July 2007 onwards), and at the neurosurgical department of the University Hospitals of Leuven. The protocol was approved by the medical ethics committees of the University of Antwerp and the University Hospitals of Leuven, and all patients provided witnessed informed consent.

### Clinical trial design

**Figure 1:**
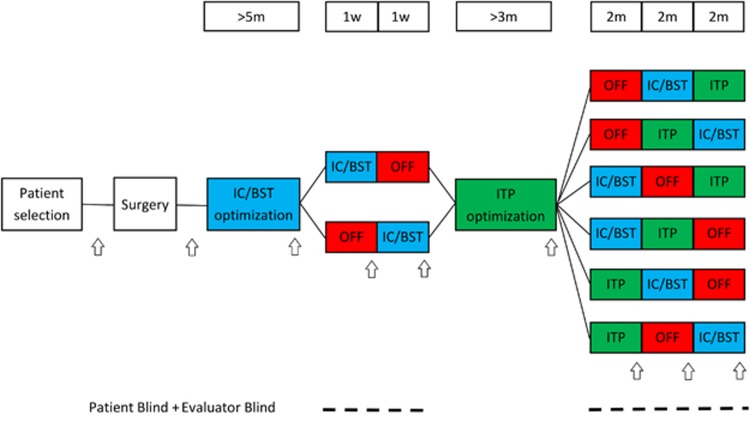
Study design. IC/BST stimulation is indicated in blue, ITP stimulation in green and stimulation OFF in red. Duration of different phases is indicated above the study design. Time points when psychiatric measures were collected, are indicated by arrows. Blinding modalities are indicated below. Evaluators (neuropsychologist and psychiatrist) and patients were blinded during the crossover phases of the study. The neurosurgeon (BN) generated the stimulation condition sequence for each patient separately (using www.random.org services) and allocated interventions accordingly. IC/BST, internal capsule/bed nucleus of stria terminalis stimulation; ITP, inferior thalamic peduncle stimulation; OFF, no stimulation; w, weeks; m, months.

For a schematic overview, see Figure 1. Four quadripolar leads (Medtronic, Minneapolis, MN, USA) were implanted; two bilaterally in IC/BST and two bilaterally in ITP. Electrical stimulation was initiated 2–4 weeks after implantation. During a subsequent period of ~5 months, stimulation parameters (voltage, pulse width, frequency and contacts used) of the anterior IC/BST electrodes were optimized. Clinical evaluation by the psychiatrist, considering both beneficial and adverse effects, was used to adjust parameters. If a stable reduction of 50% was found on HAM-D scores for a period of several weeks, patients entered the first crossover (if after a period of 1 year no stable reduction in HAM-D scores was found, patients continued to the crossover anyway). The first crossover consisted of 1 week of stimulation OFF and 1 week of IC/BST stimulation in randomized order. Our prior experience with longer (that is, 6 months) blinded crossover studies in OCD patients learned that a substantial portion of the patients might refuse to enter or drop out during such a long blinded crossover period.^22^ Therefore, the first crossover was included to optimize the chances of having an initial, brief double-blind evaluation of the hypothesized depression-reducing effects of IC/BST stimulation in as many patients as possible. After the first crossover, patients received a second optimization period, during which parameters for ITP stimulation were determined. After ITP optimization, patients continued to the second crossover. This crossover consisted of 2 months of IC/BST stimulation, 2 months of ITP stimulation and 2 months of stimulation OFF, again in a randomized order. As the change in stimulation condition at the start of each crossover phase may provoke a change in mood and potential appearance of suicidal ideation, the patient could (if necessary) be hospitalized in a psychiatric ward, without breaking the blind. An escape procedure was implemented for the second crossover, because of its longer, 2-month phases: if during one of these periods, mood deteriorated beyond baseline scores, or if both the patient and psychiatrist decided that the suffering of the patient necessitated abbreviation of that period, this was discussed in the team, and after full psychiatric evaluation, the neurosurgeon started the next condition without unblinding patient or evaluators. After the second crossover, patients could continue stimulation if they wished to do so using the target that provided the best result for the patient. Psychiatric visits were scheduled every 2 weeks during the optimization periods, and at the end of each crossover phase. If necessary, patients could request additional consults at any time throughout the study. After the crossover period, follow-up visits were planned according to clinical necessity.

### Intervention

After mounting the CRW stereotactic frame, T2-weighted magnetic resonance (MR) images without contrast medium and MPRAGE images with contrast medium were acquired together with a computed tomography (CT) of the brain. Implantation of all four electrodes was performed in a single session. Using routine stereotactic techniques under local anesthesia with sedation, the patients received quadripolar 3887-28 (patients C1–2), 3387 (C3–5) or 3391 (C6–7) leads (Medtronic) in left and right IC/BST^22, 35^ and 3389 DBS electrodes (Medtronic) in left and right ITP. The ITP target was determined using the stereotactic atlas of Mai *et al.*^36^ A postoperative CT (C4, C6 and C7) or MR scan (1.5-T system with a send/receive head radiofrequency coil only and specific absorption rate limited to 0.4 W kg^−1^) confirmed the absence of important intracranial hemorrhage and fusion with preoperative MRI allowed comparison between planned and actual trajectory of the leads. Short pulsed sequences were avoided and low specific absorption rate was used on a 1.5 T MR to preclude potentially dangerous temperature rises around the electrodes.

In C1, a 3389 lead could only be implanted in the right ITP as due to vascularization, no trajectory that safely reached the left ITP could be found. In all other patients, four leads were implanted.

### Outcome measures

Extensive well-validated and reliable measures (both clinician- and patient-rated) were taken at baseline and throughout the different stages of the study. The 17-item HAM-D^37^ was used to quantify the severity of depressive symptoms and was the primary outcome measure of this study. Response to DBS was defined as a 50% decrease on the HAM-D compared to baseline^17^ and a score of 7 or less was defined as remission.^38^ We also included secondary psychiatric measures and neuropsychological tests (for details see Supplementary Information). Evaluation time points of psychiatric outcome measures are indicated with arrows in Figure 1. Neuropsychological measures were taken at baseline and at the end of each optimization and crossover phase. After the second crossover, psychiatric outcome measures were evaluated regularly with a formal evaluation at least every 12 months including all secondary psychiatric measures.

In addition, charge densities (per phase, per contact) were evaluated for both stimulation targets.^31^ They were calculated using the contact surface area, voltage and pulse width from the second crossover and the impedances measured at the start of each crossover phase.

### Electrode position

To investigate the precise stimulation target in more detail, we transferred the center of the active contacts (cathodes) on Mai’s brain atlas.^22, 36^ Using pre- and postoperative MR and CT, we localized the exact position of the cathodes for each patient during both crossover phases and at follow-up. For C4–C6–C7, we merged preoperative MR and postoperative CT scans using Medtronic FrameLinkTM Software (Medtronic). For the remaining four patients, postoperative MR scans were used. All digital images were manually reformatted along the intercommissural plane. Finally, contact positions were determined by two observers (LL & BN) taking into account all the available neuroanatomical information and marked on the atlas plates.

### Statistical methods

The sample size for this study was calculated based upon the effects of DBS on comorbid depressive symptoms in six OCD patients.^24^ Assuming an effect size of 2 and a within-patient standard deviation of 6.5 on the HAM-D scale, a sample size of seven patients in a crossover study with two conditions (ON and OFF), results in 80% power at an alpha level of 0.05. We aimed to include 10 patients in this first exploratory trial, to account for dropout. However, the study was aborted before this original aim was reached and the final sample size during crossovers was quite small (six and five patients). The originally proposed analysis was therefore not feasible. Instead, we analyzed the primary outcome (HAM-D) using a paired *t*-test for the first crossover and follow-up periods and a repeated measures ANOVA for the second crossover, assuming the absence of carry-over effects. Normality of residuals was tested for using a Shapiro–Wilk test. Because of the limited group sizes, we refrained from using any other inferential statistics. Descriptive statistics are given for other outcome measures. IBM SPSS Statistics version 22 was used for all analyses.

## Results

### Patients

Inclusion for the study started on 1 January 2005 and ended on 31 December 2009. During these 5 years, 53 patients were screened and 11 patients were eligible. Ultimately, 4 men and 3 women were willing to participate in this study (Table 1 and Supplementary Table S1). Mean (s.d.) age at implantation was 50.0 (5.6). Participant flow is shown in Supplementary Figure S1.

Patients required intensive follow-up by the neurosurgeon and psychiatrist, with frequent patient contacts in the outpatient clinic, not only during the optimization and crossover periods, but also during follow-up. Mean number of outpatient visits to the psychiatrist (including optimization of stimulation parameters) was 21.9 per year (range 14.4–32.0). Mean number of outpatient visits to the neurosurgeon (including replacement of neurostimulators due to end of life of battery) was 4.2 per year (range 2.1–6.3) during follow-up. Patients required battery replacement on average every 14 months. To reduce the burden of these surgeries, patients were implanted with a rechargeable system (Activa RC, Medtronic).

### Outcomes

#### Postoperative period

Immediately after surgery and before neurostimulators were turned on, mean (s.d.) HAM-D dropped to 14.4 (6.7). This effect was temporary and after a period of 2–4 weeks mean (s.d.) HAM-D scores increased to 22.3 (6.8).

#### IC/BST optimization

At the end of the open optimization for the IC/BST leads, six out of seven patients were responders, and five were in remission. Stimulation parameters and HAM-D (mean (s.d.): 7.2 (3.6)) scores at the end of the IC/BST optimization period are given in Supplementary Table S2.

#### ITP optimization

Two patients aborted the ITP optimization period prematurely due to the lack of beneficial effect and were considered non-responders to DBS in ITP. They were not willing to participate in the second crossover. At the end of the open optimization for the ITP leads, four out of seven patients were responders, and one of these was in remission. Parameters and HAM-D scores (mean (s.d.): 8.8 (2.7)) at the end of the ITP optimization period are given in Supplementary Table S2.

#### Crossovers

**Figure 2:**
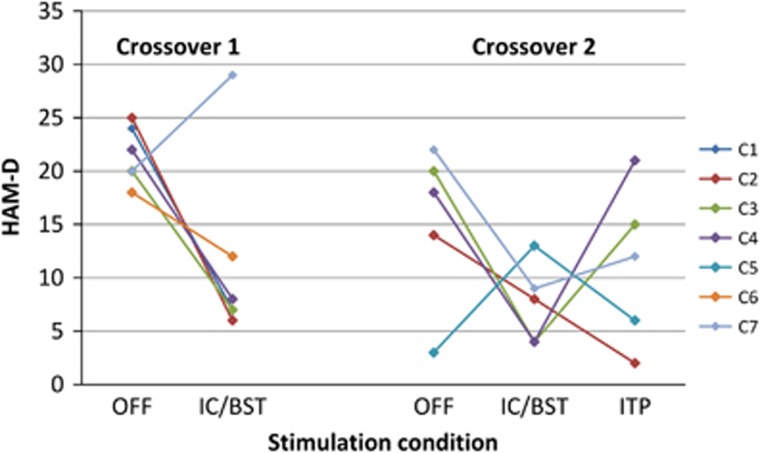
Primary outcome during crossovers. Primary outcome of both randomized crossovers, Individual data are indicated in different colors. C1–7, patients 1–7; HAM-D, Hamilton Depression Rating scale (17 items); IC/BST, internal capsule/bed nucleus of stria terminalis stimulation; ITP, inferior thalamic peduncle stimulation; OFF, no stimulation.

During the first crossover (*n*=6), mean (s.d.) HAM-D scores were 21.5 (2.7) for no stimulation (stimulator OFF) and 11.5 (8.8) for IC/BST stimulation (Figure 2). Four out of six patients were responders to DBS in IC/BST. Remission was achieved in three of those. The difference between both stimulation conditions approached significance (*t*(5)=−2.38, *P*=0.06). Five patients were able to correctly guess if they were being stimulated. Notably, C7 who showed a deterioration with DBS during the first crossover was aware of the stimulation condition.

During the second crossover (*n*=5), mean (s.d.) HAM-D scores were 15.4 (7.5) for no stimulation, 7.6 (3.8) for IC/BST stimulation and 11.2 (7.5) for ITP stimulation (Figure 2), without a main effect of stimulation condition (F_(2,8)_=1.68, *P*=0.25). During the IC/BST stimulation phase, four patients were responders to DBS, two of them being in remission. Three patients were responders to DBS in ITP and two of them were in remission. Four patients correctly guessed whether they were being stimulated, three patients were able to differentiate between ITP and IC/BST stimulation. This partial unblinding resulted from a combination of both clinical response and/or recurrence of some mild side effects which they had already experienced during the unblinded optimization periods. The stimulation OFF phase was shortened to 4 weeks in two patients because of severe deterioration of depressive symptoms. Average charge densities were calculated for ITP stimulation (42.8 μC cm^−^^2^ per phase) and IC/BST stimulation (34.3 μC cm^−^^2^ per phase) for the five patients completing both optimization periods. The difference was not statistically significant (*t*(7)=1.08, *P*=0.32).

Secondary outcomes for both crossovers are shown in Table 2. Both the Montgomery–Åsberg Depression Rating Scale (MADRS) and the Inventory of Depressive Symptoms (IDS) confirmed the decrease in depressive symptoms when stimulation was turned on. When comparing IC/BST stimulation with ITP stimulation, IC/BST generally seemed to have better effects.

Descriptive statistics for neuropsychological tests during the second crossover are summarized in Supplementary Table S3. No evidence was found for cognitive decline during stimulation in IC/BST or ITP. On the contrary, stimulation seemed to improve neuropsychological test scores. The effect was usually greatest for IC/BST stimulation. Due to the small sample size and multiple tests, we opted not to analyze these data statistically.

#### Three years and last follow-up

Three years after DBS implantation all patients were stimulated at IC/BST. Mean (s.d.) HAM-D had dropped from 24.9 (3.6) at baseline to 9.7 (5.4), that is, an average reduction of 61%. This decrease was statistically significant (*t*(6)=6.96, *P*<0.001). Five out of seven patients were responders and two were in remission. Secondary measures are shown in Supplementary Table S4.

At last follow-up, on average 63 months (range 36–97) after DBS implantation, the mean (s.d.) HAM-D was 10.6 (4.2) (significant reduction compared to baseline, *t*(6)=7.11, *P*<0.001), and only one patient (C2) preferred DBS in ITP. Secondary outcomes are shown in Supplementary Table S3. During follow-up, fluctuations in the severity of depressive symptoms were observed in most patients. Two patients committed suicide respectively after 39 and 80 months of DBS. One of these patients (C1) was a responder with a 56% decrease in HAM-D score at 79 months follow-up. The other patient (C4) had a reduction of 42% at 36 months. Four out of five remaining patients (C2, C3, C5 and C6) were responders, and two of them were in remission. C7 had a reduction of 36% at last follow-up.

Mean (s.d.) general Quality of Life Enjoyment and Satisfaction Questionnaire (Q-Les-Q) scores increased from 34.9 (9.5) to 56.0 (10.9) at last measurement (21–81 months after surgery).

Before surgery, patients took an average of 4.7 different psychotripic agents. At last follow-up, one patient required no additional medication to remain in remission. One patient only used a benzodiazepine for sleeping difficulties. On average, patients took 3.4 different psychotropic drugs.

### Electrode position

**Figure 3:**
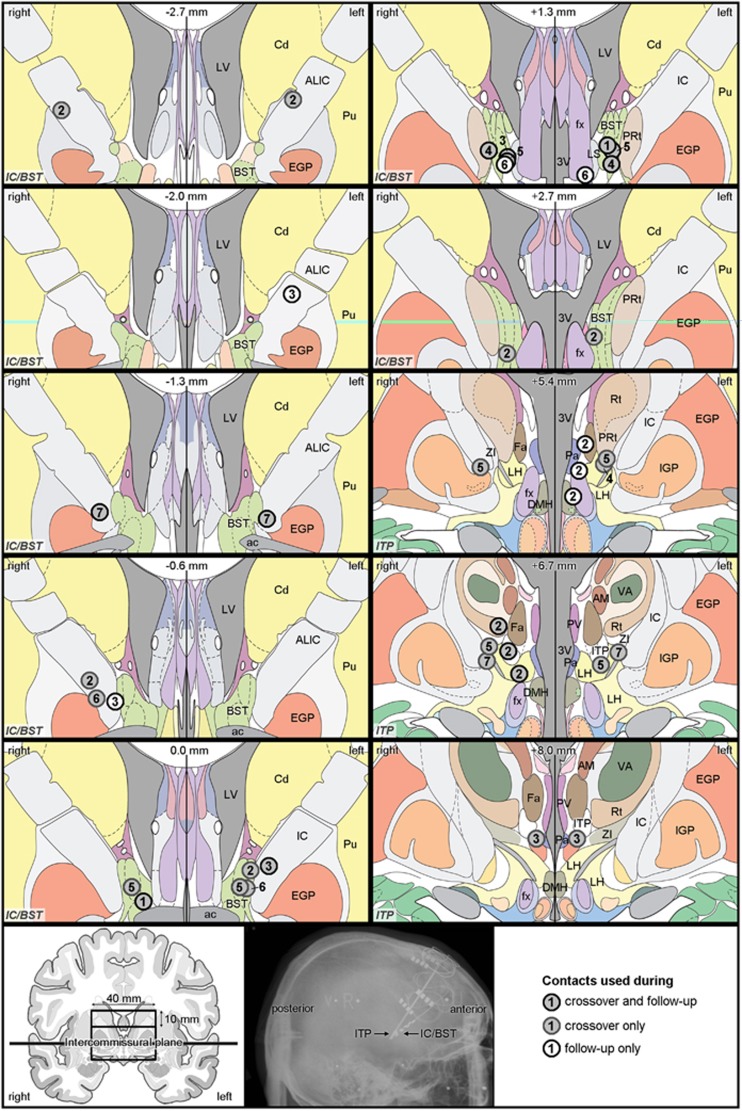
Cathode locations. Coronal brain slices showing the location of the center of cathodes selected for crossovers after the optimization periods and those used at follow-up (seven patients) (for detailed information see Supplementary Table S2). Contacts are depicted in different shades of gray, according to their usage during the crossover phase(s) and/or at follow-up, see legend in the right bottom corner. The number in each contact refers to the patient number. Note that some patients were simultaneously stimulated with more than one contact, for example, the three contacts shown on the slice at +5.4 mm are from one patient, as indicated by the repeated patient number. On each slice, the position anterior (−) or posterior (+) to the anterior commissure is specified (top center), as well as the target area (IC/BST or ITP, indicated in italic in the bottom left corner). Instead of showing complete coronal slices, a detailed window is shown, bilaterally extending 20 mm from the midline, dorsally extending 20 mm above the intercommissural plane for the IC/BST slices, and both dorsally and ventrally extending 10 mm from this plane for the ITP slices, as depicted in the bottom left panel. In addition, a sagittal radiograph of the brain with indication of the stimulation leads is shown (bottom center panel). 3V, third ventricle; ac, anterior commissure; ALIC, anterior limb of the internal capsule; AM, anteromedial thalamic nucleus; BST, bed nucleus of the stria terminalis; Cd, caudate nucleus; DMH, dorsomedial hypothalamic nucleus; EGP, external globus pallidus; Fa, fasciculosus nucleus; fx, fornix; IC, internal capsule; IGP, internal globus pallidus; ITP, inferior thalamic peduncle; LH, lateral hypothalamic area; LS, lateral septal nucleus; LV, lateral ventricle; Pa, paraventricular hypothalamic nucleus; PRt, prereticular zone; Pu, putamen; PV, paraventricular thalamic nucleus; Rt, reticular thalamic nucleus; VA, ventral anterior thalamic nucleus; ZI, zona incerta. All images, except for the radiograph, are adapted from Mai’s Atlas of the Human Brain.^36^

A detailed examination of the cathodes’ position (Figure 3) showed that, for the IC/BST target, active contacts were indeed typically located in the BST or IC. All patients had bilateral contacts in or bordering the BST during crossover and/or at follow-up. C2 and C3 were stimulated rather dorsally and anterior in the anterior limb of the IC, but they had additional active contacts, situated more ventrally in the IC and BST. For the ITP target, which is very small, three patients had cathodes centered in this brain region (C3 bilaterally, C2 and C5 unilaterally), while all other active contacts were located adjacent to the ITP.

### Adverse events

A total of 75 different adverse events (AEs) and 11 serious AEs were reported during the course of this trial. A full list can be found in Supplementary Table S5. There were four device-related serious adverse events (conversely labeled leads, two infections around neurostimulator site, damage of IC/BST electrode) leading to additional surgical procedures. The number of AEs reported during IC/BST stimulation is higher than during ITP stimulation, but the total time during which patients were stimulated in ITP is also shorter (total of 98 months versus a total of 341 months for IC/BST stimulation). Most common were psychiatric AEs (for example, increase in depressive symptoms, sleep disturbances). We noticed the emergence of extrapyramidal-like symptoms (hypomimia, micrographia, hesitant walking, less fluent movement) during ITP stimulation in one patient, which disappeared when stimulation was turned off. We have not yet encountered such symptoms in our experience with DBS in IC/BST.

An increase in depressive symptoms was reported by all patients at some point. Some patients also reported an increase of suicidal thoughts. One patient (C5) showed, after a period of remission, a deterioration of depressive symptoms and was hospitalized. During this hospitalization, he committed suicide, 39 months after DBS implantation. No new stressful life events were mentioned in the period preceding the suicide. Another patient (C1) responded well to DBS but had frequent relapses with suicidal ideations and suicide attempts. After 79 months of stimulation, the patient was a responder to DBS, but committed suicide a month after the last evaluation. Both patients had a history of suicide attempts before DBS implantation. Note that CT scans were performed to evaluate a potential electrode position shift in these patients (respectively 34 and 51 months after electrode implantation), but no electrode deviation was found.

## Discussion

In this study, two stimulation targets were compared directly, to our knowledge for the first time in a psychiatric population. Although we clearly observed clinically relevant effects in our patients, predominantly for stimulation in IC/BST, these findings could not be substantiated statistically in the crossovers, probably due to a lack of power, given the limited sample size, which is the main limitation of this study. Therefore the data and conclusions presented here, have to be considered as being preliminary. Another limitation in this study is partial unblinding, as blinded patients and psychiatrists were often able to correctly guess whether stimulation was ON or OFF. There was, however, less confidence about the target that was being stimulated.

The second crossover did not give a decisive answer regarding the superiority of either IC/BST stimulation or ITP stimulation, but the last follow-up data might provide some clues, as six out of seven patients preferred IC/BST stimulation. After completion of both crossovers, only one patient (C2) preferred further ITP over IC/BST stimulation. At last follow-up, that is, 8 years after implantation, she continued to experience a substantial decrease in depressive symptoms as compared to the preoperative situation (HAM-D reduction from 31 to 6). Note that the charge densities for both targets were similar and can therefore not account for potential differences in outcome per target. This finding also refutes our initial hypothesis that ITP stimulation might require lower charge densities than IC/BST.

It has been suggested that differentiation among symptom clusters of depression might guide the choice between stimulation targets.^39^ In our study, we could not detect differences in the phenomenology of MDD in the only patient that responded better to ITP stimulation. She experienced a traumatic childhood and had a secondary diagnosis of an anxiety disorder, but was not the only patient with these characteristics. Her anxiety diminished markedly under ITP stimulation and less under IC/BST stimulation. The other two patients with a secondary diagnosis of an anxiety disorder responded better to IC/BST stimulation, with a substantial reduction in HAM-A scores, consistent with our findings in OCD patients.^22^

Careful analysis of the neuroanatomical position of the active electrode contacts indicated that all patients were stimulated bilaterally in or bordering the targeted structure (note that the stimulated volume extends further that physical boundaries of the contact^22^), sometimes with additional contacts outside this region. Variability in clinical outcome of DBS in this target can therefore not be explained solely by the anatomical position of the cathodes. This is in line with previous research in another DBS target for TRD (subcallosal cingulate), showing that the contact location was not the only factor discriminating responders from non-responders,^40^ and with our own findings with DBS for OCD in BST.^22^ Despite the absence of statistical significance in the crossovers, at the time of writing, four patients continue to receive IC/BST stimulation and experience substantial alleviation of depressive symptoms.

IC/BST and ITP stimulation were accompanied by similar adverse events, and after the crossovers, the IC/BST target was generally chosen on account of better clinical effects, not because of fewer (or less severe) adverse events. Neuropsychological test scores did not reveal any acute worsening of cognitive function during either IC/BST or ITP stimulation, which is in line with previous DBS studies.^22, 41^ As most severe adverse events, we unfortunately report that two patients committed suicide during the course of this study. Patients suffering from severe depression are already at high risk for committing suicide (up to 15% [^ref. 42^]). A patient not responding to DBS might lose any hope of recovery, as DBS is often regarded as a last resort. Nevertheless, even patients who do respond to DBS may continue to be at risk for suicide, for example, in case of (temporary) relapses. Moreover, suicidal ideation might persist even when treatment reduces depressive symptoms in general.^42^ Other DBS for TRD trials have also reported completed suicides,^12, 43^ although suicide rates remained below those of severe MDD patients without DBS.^44^ The intense follow-up provided after DBS implantation might protect against suicide. However, it has been put forward that DBS might increase impulsivity,^45^ which could lower the threshold for suicide attempts in DBS patients.^46^ In our study, the suicides did not seem to be related to DBS, nor were there any signs of increased impulsivity in these patients.

Although we did not include new patients after the second suicide, these unfortunate occurrences were not the deciding factor for the premature end of this study. Rather, recruitment of patients was much slower than expected (only seven patients in 5 years) and implantation in ITP did not seem to have added beneficial effects in most patients, questioning the justification of continuing with a more complicated intervention, with four electrodes instead of two. Although our within-subject approach allows for the most scientifically sound comparison of different DBS targets, the lengthy study design might discourage patients, as it requires even more commitment than a ‘regular’ DBS crossover study with only one target. Taken together, a within-subject evaluation of different DBS targets remains very challenging.

Open-label trials in SCC, NAc and VC/VS generally found that about half of the TRD patients responded to the treatment,^12, 13, 14, 15, 16, 17, 18^ similar to our findings at last follow-up, but response rates might be even higher for DBS in the supero-lateral MFB, with six out of seven responders in a pilot study.^20^ As mentioned above, different targets might influence different symptom clusters, while resulting in comparable overall scores on depression rating scales, explaining the comparability of results in open-label studies. Electrical stimulation of the NAc, for example, reduces anhedonia.^47^ Stimulation in SCC appears to first improve mood and only later are sleep disturbances and anxiety symptoms observed.^48^ In a rat model for depression, DBS in the MFB and SCC analog seemed to alleviate different symptoms.^49^

A randomized controlled trial of DBS in VC/VS did not find a significant difference between an active and sham-stimulation group.^19^ A recent multicenter crossover study of DBS in the anterior limb of IC had more promising results with a statistically significant reduction of depressive symptoms during active stimulation as compared to no stimulation.^50^ This seems to be in line with our preliminary results of stimulation in IC/BST.

In conclusion, we found a clinically relevant effect of DBS on MDD symptoms in severe treatment-refractory patients for both IC/BST and ITP stimulation. These observations could not be statistically confirmed during the crossover phases of this study, probably due to the limited sample size. We do find a statistically significant reduction in depressive symptoms when comparing baseline with 3-year follow-up and with last follow-up (3–8 months). All patients, except one, preferred IC/BST stimulation.

## Figures and Tables

**Table 1:** Demographic data

*Patient*	*Gender*	*Age at onset (years)*	*Age at implant (years)*	*Primary diagnosis*	*Secondary diagnosis (axes I and II)*	*Illness duration (years)*	*HAM-D at baseline*
C1	F	32	43	MDD	Anxiety disorder NOS, schizotypal PD	11	27
C2	F	47	52	MDD	Panic disorder, specific phobia	5	31
C3	M	29	45	MDD	Dependent PD	16	23
C4	M	37	47	MDD	Generalized anxiety disorder, panic disorder	10	26
C5	M	45	56	MDD	—	11	21
C6	M	22	58	MDD	Obsessive-compulsive PD	36	21
C7	F	35	49	MDD	Dependent PD	14	25

Abbreviations: C1–7, patients 1–7; HAM-D, Hamilton Depression Rating scale (17-items); MDD, major depressive disorder; NOS, not otherwise specified; PD, personality disorder Demographic data for all seven patients at baseline. Primary and secondary diagnoses according to DSM-IV-TR criteria. Illness duration refers to the time between first emergence of depressive symptoms and DBS implantation.

**Table 2:** Secondary measures during crossovers

*Scale*	*Crossover 1 (*n=*6)*		*Crossover 2 (*n=*5)*		
	*OFF mean (s.d.)*	*IC/BST mean (s.d.)*	*OFF mean (s.d.)*	*IC/BST mean (s.d)*	*ITP mean (s.d.)*
MADRS	36.0 (3.9)	16.5 (11.7)	25.0 (9.6)	13.4 (6.9)	17.6 (9.7)
IDS	40.0 (10.7)	31.0 (16.9)	35.0 (8.9)	16.6 (5.8)	33.2 (15.8)
BHS	14.3 (6.0)	11.7 (6.1)	12.6 (4.3)	6.4 (2.7)	10.0 (4.1)
HAM-A	17.5 (3.2)	11.0 (9.5)	10.8 (4.0)	7.4 (3.4)	7.2 (4.7)
GAF	47.5 (5.2)	60.8 (11.1)	52.0 (12.5)	68.0 (8.4)	62.0 (16.4)
CGI-S	4.7 (0.5)	2.3 (1.5)	3.2 (1.6)	1.4 (1.1)	2.6 (1.5)
PGI-S	5.2 (0.4)	2.5 (2.1)	3.8 (1.8)	1.8 (1.6)	2.6 (1.8)
CGI-I	3.2 (0.4)	4.8 (1.2)	3.8 (1.3)	5.0 (1.0)	4.6 (1.0)
PGI-I	3.0 (0.0)	4.8 (1.2)	3.8 (0.8)	4.8 (0.8)	1.6 (1.1)
SCL-90			184.0 (51.4)	145.6 (46.1)	159.0 (45.6)

Abbreviations: BHS, Beck Hopelessness Scale; CGI-I, Clinical Global Impressions-Improvement; CGI-S, clinical global impressions-severity; GAF, global assessment of function; HAM-A, Hamilton Anxiety Rating Scale; IC/BST, internal capsule/bed nucleus of stria terminalis stimulation; IDS, Inventory for Depressive Symptoms; ITP, inferior thalamic peduncle stimulation; MADRS, Montgomery–Åsberg Depression Rating Scale; OFF, no stimulation; PGI-I, patient global impressions-improvement; PGI-S, patient global impressions-severity; SCL-90, symptom checklist 90 items. Summary of secondary measures during both crossovers. Means and s.d. are shown.

## References

[bib1] Sobocki P, Jönsson B, Angst J, Rehnberg C. Cost of depression in Europe. J Ment Health Policy Econ 2006; 9: 87–98. 17007486

[bib2] Murray CJL, Vos T, Lozano R, Naghavi M, Flaxman AD, Michaud C et al. Disability-adjusted life years (DALYs) for 291 diseases and injuries in 21 regions, 1990-2010: a systematic analysis for the Global Burden of Disease Study 2010. Lancet 2012; 380: 2197–2223. 23245608 10.1016/S0140-6736(12)61689-4

[bib3] Kessler RC, Berglund P, Demler O, Jin R, Merikangas KR, Walters EE. Lifetime prevalence and age-of-onset distributions of DSM-IV disorders in the National Comorbidity Survey Replication. Arch Gen Psychiatry 2005; 62: 593–602. 15939837 10.1001/archpsyc.62.6.593

[bib4] Angst J, Paksarian D, Cui L, Merikangas KR, Hengartner MP, Ajdacic-Gross V et al. The epidemiology of common mental disorders from age 20 to 50: results from the prospective Zurich cohort Study. Epidemiol Psychiatr Sci 2015; 25: 24–32. 25802979 10.1017/S204579601500027X PMC6998675

[bib5] Rush J, Trivedi MH, Wisniewski SR, Nierenberg A, Stewart JW, Warden D et al. Acute and longer-term outcomes in depressed outpatients requiring one or several treatment steps: a STAR*D report. Am J Psychiatry 2006; 163: 1905–1917. 17074942 10.1176/ajp.2006.163.11.1905

[bib6] Cuijpers P, Karyotaki E, Weitz E, Andersson G, Hollon SD, van Straten A. The effects of psychotherapies for major depression in adults on remission, recovery and improvement: a meta-analysis. J Affect Disord 2014; 159: 118–126. 24679399 10.1016/j.jad.2014.02.026

[bib7] Slotema CW, Blom JD, Hoek HW, Sommer IEC. Should we expand the toolbox of psychiatric treatment methods to include Repetitive Transcranial Magnetic Stimulation (rTMS)? A meta-analysis of the efficacy of rTMS in psychiatric disorders. J Clin Psychiatry 2010; 71: 873–884. 20361902 10.4088/JCP.08m04872gre

[bib8] Pagnin D, de Queiroz V, Pini S, Cassano GB. Efficacy of ECT in depression: a meta-analytic review. J ECT 2004; 20: 13–20. 15087991 10.1097/00124509-200403000-00004

[bib9] Solomon D, Keller MB, Leon C, Mueller TI, Lavori PW, Shea MT et al. Multiple recurrences of major depressive disorder. Am J Psychiatry 2000; 157: 229–233. 10671391 10.1176/appi.ajp.157.2.229

[bib10] Eaton WW, Shao H, Nestadt G, Lee HB, Bienvenu OJ, Zandi P. Population-based study of first onset and chronicity in major depressive disorder. Arch Gen Psychiatry 2008; 65: 513–520. 18458203 10.1001/archpsyc.65.5.513 PMC2761826

[bib11] Drevets WC, Price JL, Furey ML. Brain structural and functional abnormalities in mood disorders: implications for neurocircuitry models of depression. Brain Struct Funct 2008; 213: 93–118. 18704495 10.1007/s00429-008-0189-x PMC2522333

[bib12] Kennedy SH, Giacobbe P, Rizvi SJ, Placenza FM, Nishikawa Y, Mayberg HS et al. Deep brain stimulation for treatment-resistant depression: follow-up after 3 to 6 years. Am J Psychiatry 2011; 168: 502–510. 21285143 10.1176/appi.ajp.2010.10081187

[bib13] Holtzheimer PE, Kelley ME, Gross RE, Filkowski MM, Kozarsky J, Chismar R et al. Subcallosal cingulate deep brain stimulation for treatment-resistant unipolar and bipolar depression. Arch Gen Psychiatry 2014; 69: 150–158. 10.1001/archgenpsychiatry.2011.1456 PMC4423545 22213770

[bib14] Ramasubbu R, Anderson S, Haffenden A, Chavda S, Kiss ZHT. Double-blind optimization of subcallosal cingulate deep brain stimulation for treatment-resistant depression: a pilot study. J Psychiatry Neurosci 2013; 38: 325–332. 23527884 10.1503/jpn.120160 PMC3756116

[bib15] Puigdemont D, Pérez-Egea R, Portella MJ, Molet J, de Diego-Adeliño J, Gironell A et al. Deep brain stimulation of the subcallosal cingulate gyrus: further evidence in treatment-resistant major depression. Int J Neuropsychopharmacol 2012; 15: 121–133. 21777510 10.1017/S1461145711001088

[bib16] Lozano AM, Giacobbe P, Hamani C, Rizvi SJ, Kennedy SH, Kolivakis TT et al. A multicenter pilot study of subcallosal cingulate area deep brain stimulation for treatment-resistant depression. J Neurosurg 2012; 116: 315–322. 22098195 10.3171/2011.10.JNS102122

[bib17] Bewernick BH, Hurlemann R, Matusch A, Kayser S, Grubert C, Hadrysiewicz B et al. Nucleus accumbens deep brain stimulation decreases ratings of depression and anxiety in treatment-resistant depression. Biol Psychiatry 2010; 67: 110–116. 19914605 10.1016/j.biopsych.2009.09.013

[bib18] Malone D, Dougherty DD, Rezai AR, Carpenter LL, Friehs GM, Eskandar EN et al. Deep brain stimulation of the ventral capsule/ventral striatum for treatment-resistant depression. Biol Psychiatry 2009; 65: 267–275. 18842257 10.1016/j.biopsych.2008.08.029 PMC3486635

[bib19] Dougherty DD, Rezai AR, Carpenter LL, Howland RH, Bhati MT, O’Reardon JP et al. A randomized sham-controlled trial of deep brain stimulation of the ventral capsule/ventral striatum for chronic treatment-resistant depression. Biol Psychiatry 2015; 78: 240–248. 25726497 10.1016/j.biopsych.2014.11.023

[bib20] Schlaepfer TE, Bewernick BH, Kayser S, Burkhard M, Coenen VA. Rapid effects of deep brain stimulation for treatment-resistant major depression. Biol Psychiatry 2013; 73: 1204–1212. 23562618 10.1016/j.biopsych.2013.01.034

[bib21] Nuttin B, Cosyns P, Demeulemeester H, Gybels J, Meyerson B. Electrical stimulation in anterior limbs of internal capsules in patients with obsessive-compulsive disorder. Lancet 1999; 354: 1526. 10.1016/S0140-6736(99)02376-4 10551504

[bib22] Luyten L, Hendrickx S, Raymaekers S, Gabriëls L, Nuttin B. Electrical stimulation in the bed nucleus of the stria terminalis alleviates severe obsessive-compulsive disorder. Mol Psychiatry 2016; 21: 1272–1280. 26303665 10.1038/mp.2015.124

[bib23] Gabriëls L, Cosyns P, Nuttin B, Demeulemeester H, Gybels J. Deep brain stimulation for treatment-refractory obsessive-compulsive disorder: psychopathological and neuropsychological outcome in three cases. Acta Psychiatr Scand 2003; 107: 275–282. 12662250

[bib24] Nuttin BJ, Gabriëls L, Cosyns PR, Meyerson B, Andréewitch S, Sunaert SG et al. Long-term electrical capsular stimulation in patients with obsessive-compulsive disorder. Neurosurgery 2003; 52: 1263–1274. 12762871 10.1227/01.neu.0000064565.49299.9a

[bib25] Luyck K, Luyten L. Can electrical stimulation of the human bed nucleus of the stria terminalis reduce contextual anxiety? An unanswered question. Front Behav Neurosci 2015; 9: 69. 25852509 10.3389/fnbeh.2015.00069 PMC4362315

[bib26] Luyten L, Casteels C, Vansteenwegen D, van Kuyck K, Koole M, Van Laere K et al. Micro-positron emission tomography imaging of rat brain metabolism during expression of contextual conditioning. J Neurosci 2012; 32: 254–263. 22219287 10.1523/JNEUROSCI.3701-11.2012 PMC6621336

[bib27] Davis M, Walker DL, Miles L, Grillon C. Phasic vs sustained fear in rats and humans: role of the extended amygdala in fear vs anxiety. Neuropsychopharmacology 2010; 35: 105–135. 19693004 10.1038/npp.2009.109 PMC2795099

[bib28] Dong H-W, Swanson LW. Projections from bed nuclei of the stria terminalis, anteromedial area: cerebral hemisphere integration of neuroendocrine, autonomic, and behavioral aspects of energy balance. J Comp Neurol 2006; 494: 142–178. 16304685 10.1002/cne.20788 PMC2563961

[bib29] Crestani C, Alves F, Gomes F, Resstel L, Correa F, Herman J. Mechanisms in the bed nucleus of the stria terminalis involved in control of autonomic and neuroendocrine functions: a review. Curr Neuropharmacol 2013; 11: 141–159. 23997750 10.2174/1570159X11311020002 PMC3637669

[bib30] Neumann W, Huebl J, Brücke C, Gabriëls L, Bajbouj M, Merkl A et al. Different patterns of local field potentials from limbic DBS targets in patients with major depressive and obsessive compulsive disorder. Mol Psychiatry 2014; 19: 1186–1192. 24514569 10.1038/mp.2014.2 PMC4813757

[bib31] Fakhar K, Hastings E, Butson CR, Foote KD, Zeilman P, Okun MS. Management of deep brain stimulator battery failure: battery estimators, charge density, and importance of clinical symptoms. PLoS ONE 2013; 8: e58665. 23536810 10.1371/journal.pone.0058665 PMC3594176

[bib32] Jiménez F, Velasco F, Salin-Pascual R, Hernandez J, Velasco M, Criales J et al. A patient with a resistant major depression disorder treated with deep brain stimulation in the inferior thalamic peduncle. Neurosurgery 2005; 57: 585–593. 16145540 10.1227/01.neu.0000170434.44335.19

[bib33] Velasco F, Velasco M, Jiménez F, Velasco AL, Salin-Pascual R. Neurobiological background for performing surgical intervention in the inferior thalamic peduncle for treatment of major depression disorders. Neurosurgery 2005; 57: 439–448, discussion 439–48. 16145522 10.1227/01.neu.0000172172.51818.51

[bib34] First M, Spitzer R, Gibbon M, Williams J Structured Clinical Interview for DSM-IV Axis I Disorders, Patient Edition. Biometrics Research, New York State Psychiatric Institut: New York, 1995.

[bib35] Nuttin B, Gielen F, van Kuyck K, Wu H, Luyten L, Welkenhuysen M et al. Targeting bed nucleus of the stria terminalis for severe obsessive-compulsive disorder: more unexpected lead placement in obsessive-compulsive disorder than in surgery for movement disorders. World Neurosurg 2013; 80(S30): e11–e16. 10.1016/j.wneu.2012.12.029 23268197

[bib36] Mai JK, Paxinos G, Voss T. Atlas of the Human Brain. Third Edition, Academic Press: San Diego, 2007.

[bib37] Hamilton M. Rating depressive patients. J Clin Psychiatry 1980; 41: 21–24. 7440521

[bib38] Zimmerman M, Chelminski I, Posternak M. A review of studies of the Hamilton depression rating scale in healthy controls: implications for the definition of remission in treatment studies of depression. J Nerv Ment Dis 2004; 192: 595–601. 15348975 10.1097/01.nmd.0000138226.22761.39

[bib39] Williams NR, Okun MS. Deep brain stimulation (DBS) at the interface of neurology and psychiatry. J Clin Invest 2013; 123: 4546–4556. 24177464 10.1172/JCI68341 PMC3809784

[bib40] Riva-Posse P, Choi KS, Holtzheimer PE, McIntyre CC, Gross RE, Chaturvedi A et al. Defining critical white matter pathways mediating successful subcallosal cingulate deep brain stimulation for treatment-resistant depression. Biol Psychiatry 2014; 76: 963–969. 24832866 10.1016/j.biopsych.2014.03.029 PMC4487804

[bib41] Kubu C, Malone D, Chelune G, Malloy P, Rezai A, Frazier T et al. Neuropsychological outcome after deep brain stimulation in the ventral capsule/ventral striatum for highly refractory obsessive-compulsive disorder or major depression. Stereotact Funct Neurosurg 2013; 9: 374–378. 10.1159/000348321 24108099

[bib42] Seo H-J, Jung Y-E, Jeong S, Kim J-B, Lee M-S, Kim J-M et al. Persistence and resolution of suicidal ideation during treatment of depression in patients with significant suicidality at the beginning of treatment: the CRESCEND study. J Affect Disord 2014; 155: 208–215. 24262641 10.1016/j.jad.2013.11.002

[bib43] Bewernick BH, Kayser S, Sturm V, Schlaepfer TE. Long-term effects of nucleus accumbens deep brain stimulation in treatment-resistant depression: evidence for sustained efficacy. Neuropsychopharmacology 2012; 37: 1975–1985. 22473055 10.1038/npp.2012.44 PMC3398749

[bib44] Wulsin L, Vaillant G, Wells VE. A systematic review of the mortality of depression. Psychosom Med 1999; 61: 6–17. 10024062 10.1097/00006842-199901000-00003

[bib45] Luigjes J, Mantione M, van den Brink W, Schuurman PR, van den Munckhof P, Denys D. Deep brain stimulation increases impulsivity in two patients with obsessive-compulsive disorder. Int Clin Psychopharmacol 2011; 26: 338–340. 21857527 10.1097/YIC.0b013e32834af505

[bib46] Turecki G. Dissecting the suicide phenotype: the role of impulsive-aggressive behaviours. J Psychiatry Neurosci 2005; 30: 398–408. 16327873 PMC1277022

[bib47] Schlaepfer TE, Cohen MX, Frick C, Kosel M, Brodesser D, Axmacher N et al. Deep brain stimulation to reward circuitry alleviates anhedonia in refractory major depression. Neuropsychopharmacology 2008; 33: 368–377. 17429407 10.1038/sj.npp.1301408

[bib48] Lozano AM, Mayberg HS, Giacobbe P, Hamani C, Craddock RC, Kennedy SH. Subcallosal cingulate gyrus deep brain stimulation for treatment-resistant depression. Biol Psychiatry 2008; 64: 461–467. 18639234 10.1016/j.biopsych.2008.05.034

[bib49] Edemann-Callesen H, Voget M, Empl L, Vogel M, Wieske F, Rummel J et al. Medial forebrain bundle deep brain stimulation has symptom-specific anti-depressant effects in rats and as opposed to ventromedial prefrontal cortex stimulation interacts with the reward system. Brain Stimul 2015; 8: 714–723. 25819024 10.1016/j.brs.2015.02.009

[bib50] Bergfeld IO, Mantione M, Hoogendoorn MLC, Ruhé HG, Notten P, van Laarhoven J et al. Deep brain stimulation of the ventral anterior limb of the internal capsule for treatment-resistant depression. JAMA Psychiatry 2016; 73: 456. 27049915 10.1001/jamapsychiatry.2016.0152

