# Supplementary material for: Deep brain stimulation for treatment-resistant major depressive disorder: a comparison of two targets and long-term follow-up
Source: Transl Psychiatry. 2017 Oct 31;7(10):e1251–. doi: 10.1038/tp.2017.66 (PMC5682606; doi:10.1038/tp.2017.66)
Supplement: Supplementary Information [file tp201766x1.docx]

**Supplementary information**

**Deep Brain Stimulation for Treatment-Resistant Major Depressive Disorder:
a Comparison of Two Targets and Long-Term Follow-Up**

**Simon Raymaekers*, Laura Luyten*, Chris Bervoets, Loes Gabriëls, Bart Nuttin**

*Both authors contributed equally

1. **Supplementary eligibility criteria**

Treatment history was required to prove failure in response to: 1) adequate trials of primary antidepressants from at least 3 different classes for more than 6 weeks at the maximum recommended or tolerated dose AND 2) adequate trials of augmentation or combination of a primary antidepressant using at least 2 different augmenting or combination agents, e.g., lithium, T3, stimulants, neuroleptics, anticonvulsants, buspirone, or a second primary antidepressant, for more than four weeks at the usually recommended or maximum tolerated dose AND 3) an adequate trial of ECT: more than six bilateral treatments AND 4) adequate relapse prevention by antidepressant medication or maintenance ECT or an adequate trial of individual psychotherapy. Patients had to be able to provide informed consent, and to comply with the operational and administrative requirements of participation in the study and with the questionnaires and the protocol. They had to be in good general health, and drug-free or on a stable drug regimen for at least 6 weeks at the time of entry into the study.

Patients were not allowed to enter the study if there was a current or past non-affective psychotic disorder, current or unstably remitted substance abuse (caffeine and tobacco were allowed), any current clinically significant neurological disorder or medical illness affecting brain function. Further exclusion criteria were any clinically significant abnormality on preoperative magnetic resonance imaging (MRI), any surgical contraindications to undergo DBS, including labeled contraindications for DBS, inability to undergo an awake operation and/or inability to undergo preoperative MRI. Also patients with infection, coagulopathy, or significant cardiac or other medical risk factors for surgery were excluded. Pregnant women and women of childbearing age, not using effective contraception, were excluded. Patients with a history of severe personality disorder, especially cluster B or with imminent risk of suicide (based on the judgment of the investigators) were also excluded.

1. **Secondary Measures**

Besides the 17 item HAM-D ^1^, we used the Montgomery–Åsberg Depression Rating Scale ^2^, and the Inventory for Depressive Symptoms, Self-Report (IDS-SR), a comprehensive patient-rated measure ^3^ to evaluate depressive symptoms. To quantify hopelessness, the Beck Hopelessness Scale (BHS) ^4^ was scored. Anxiety was measured with the Hamilton Anxiety Rating scale (HAM-A) ^5^. Manic symptoms were measured using the Young Mania Rating scale (YMRS) ^6^. Global measures of illness severity and improvement included the Clinical Global Impressions - Severity (CGI-S) and Clinical Global Impressions - Improvement (CGI-I) scales, and the Patient Global Impressions (PGI-S and PGI-I) scale ^7^. Other measures of psychiatric disability were the clinician-rated Global Assessment of Function (GAF) ^8^. Quality of life was evaluated using the patient-rated Quality of Life Enjoyment and Satisfaction Questionnaire (Q-LES-Q) ^9^, with scores calculated as percentages. The patient-rated Symptom CheckList (SCL-90) ^10^ screened for a broad range of psychopathological symptoms.

Neuropsychological tests were done at baseline (2-4 weeks before surgery) and at the end of each phase of the triple crossover trial. Practice effects were minimized by the use of parallel test forms (when available). The following cognitive tests were used: Rey Auditory Verbal Learning Test (RAVLT), Trail Making Test A & B (TMT A & B), Stroop Test, Rey-Osterrieth Complex Figure Test (RO-CFT), Standard Progressive Matrices (SPM) of Raven, Digit Span Test (DSpT), Word Fluency Test (WFT).

References for psychiatric scales:

1 Hamilton M. Rating Scale for Depression. *J Neurol Neurosurg Psychiatry* 1960; **23**: 56–61.

2 Montgomery S, Asberg M. A new depression scale designed to be sensitive to change. *Br J Psychiatry* 1979; **Apr**: 382–9.

3 Rush A, Giles D, Schlesser M, Fulton C, Weissenburger J, Burns C. The Inventory for Depressive Symptomatology (IDS): preliminary findings. *Psychiatry Res* 1986; **18**: 65–87.

4 Beck A. Beck Hopelessness Scale. *Psychol Corp* 1988.

5 Hamilton M. The assesment of anxiety states by rating. *Br J Med Psychol* 1959; **32**: 50–55.

6 Young RC, Biggs JT, Ziegler VE, Meyer DA. A rating scale for mania: reliability, validity and sensitivity. *Br J Psychiatry* 1978; **133**: 429–35.

7 Guy W. Clinical Global Impressions. Rockville, 1976.

8 Hall R. Global Assessment of Functioning. A modified scale. *Psychosomatics* 1995; **36**: 267–275.

9 Endicott J, Harrison W, Blumenthal R. Quality of Life Enjoyment and Satisfaction Questionnaire: a new measure. *Psychopharmacol Bull* 1993; **29**: 321–326.

10 Derogatis LR. Symptom Checklist-90-Revised (SCL-90-R). *Pearson Educ Inc, San Antonio* 1993.

1. **Supplementary figures and tables**

4 declined to participate

7 patients were implanted with electrodes in IC/BST and ITP

11 patients were repeatedly informed about procedure and protocol

53 patients were assessed for eligibility

53 patients were assessed for eligibility

42 were not eligible:

10 showed bipolarity on SCID-IV

9 did not have ECT

4 had cognitive deficits and were unable to sign informed consent

7 abused benzodiazepines

4 abused amphetamines

8 had cluster B personality disorder with high risk of acting out

1 patient had conversely labelled leads and was stimulated in ITP instead of IC/BST – data from first crossover were discarded

6 patients completed the IC/BST optimization period and the first randomized crossover

2 patients chose not to participate in the second crossover

5 patients completed ITP optimization and second randomized crossover

**Figure S1: Participant flow**

SCID-IV: Structured clinical interview for DSM-IV; ECT: Electroconvulsion therapy; IC/BST: internal capsule/bed nucleus of stria terminalis; ITP: inferior thalamic peduncle

| **Patient** | **SSRI** | **SNRI** | **TCA** | **MAO-I** | **NRI** | **TeCA** | **Other AD** | **BZD** | **NA** | **OA** | **VN** | **Hosp** |
| --- | --- | --- | --- | --- | --- | --- | --- | --- | --- | --- | --- | --- |
| C1 | 2 | 1 | 3 | 2 | 2 | 1 | 2 | 5 | 4 | 5 | 0 | >650 |
| C2 | 2 | 1 | 1 | 1 | 0 | 1 | 2 | 6 | 7 | 3 | 0 | >400 |
| C3 | 3 | 0 | 2 | 0 | 0 | 0 | 2 | 2 | 5 | 1 | 0 | >700 |
| C4 | 3 | 2 | 4 | 1 | 0 | 0 | 1 | 3 | 6 | 1 | 1 | >200 |
| C5 | 2 | 1 | 2 | 1 | 0 | 1 | 1 | 0 | 2 | 2 | 0 | 30? |
| C6 | 2 | 0 | 3 | 2 | 1 | 2 | 3 | 4 | 2 | 2 | 0 | >300 |
| C7 | 2 | 0 | 2 | 1 | 0 | 0 | 2 | 3 | 6 | 1 | 1 | >300 |

**Table S1: Patient treatment history**. Number of medications of specific classes prescribed to patient before inclusion in the study and approximate number of days spent in hospital due to MDD (Hosp). SSRI: selective serotonin reuptake inhibitor; SNRI: serotonin and noradrenalin reuptake inhibitor; TCA: tricyclic antidepressant; MAO-I: monoamine oxidase inhibitor; NRI: noradrenalin reuptake inhibitor; TeCA: tetracyclic antidepressant; other AD: other antidepressants; BZD: benzodiazepine, NA: neuroleptic augmentation, OA: other augmentation, VN: vagus nerve stimulator

| **Patient** | **Crossover IC/BST left** | | | | **Crossover IC/BST right** | | | | **HAM-D** |  |
| --- | --- | --- | --- | --- | --- | --- | --- | --- | --- | --- |
|  | **Contacts** | **Voltage** | **PW** | **Freq** | **Contacts** | **Voltage** | **PW** | **Freq** |  |  |
| C1 | 0-c+ | 9 | 90 | 130 | 0-c+ | 8 | 60 | 130 | 5 |  |
| C2 | 0-1-2-c+ | 4.5 | 210 | 130 | 0-1-2-c+ | 4.5 | 210 | 130 | 4 |  |
| C3 | 1-2+ | 8.5 | 300 | 130 | 1-2+ | 8.5 | 210 | 130 | 6 |  |
| C4 | 1-c+ | 5.5 | 210 | 130 | 1-c+ | 5.5 | 210 | 130 | 6 |  |
| C5 |  |  |  |  |  |  |  |  |  |  |
| C6 | 0+1- | 6 | 240 | 130 | 0+1- | 6 | 240 | 130 | 8 |  |
| C7 | 1-c+ | 7.5 | 210 | 130 | 1-c+ | 7.5 | 210 | 130 | 14 |  |
| **Patient** | **Crossover ITP left** | | | | **Crossover ITP right** | | | | **HAM-D** |  |
|  | **Contacts** | **Voltage** | **PW** | **Freq** | **Contacts** | **Voltage** | **PW** | **Freq** |  |  |
| C1 |  |  |  |  |  |  |  |  |  |  |
| C2 |  |  |  |  | 0-1-2-c+ | 4 | 210 | 130 | 6 |  |
| C3 | 0-1+ | 8.2 | 300 | 130 | 0-1+ | 8.2 | 300 | 130 | 9 |  |
| C4 | 1-2+ | 4.5 | 210 | 130 |  |  |  |  | 13 |  |
| C5 | 0+1-2-3+ | 7 | 300 | 130 | 0+1-2-3+ | 7 | 300 | 130 | 7 |  |
| C6 |  |  |  |  |  |  |  |  |  |  |
| C7 | 3-c+ | 4.5 | 300 | 130 | 3-c+ | 4.5 | 300 | 130 | 9 |  |
| **Patient** | **Final left** | | | | **Final right** | | | | **HAM-D** | **Final target** |
|  | **Contacts** | **Voltage** | **PW** | **Freq** | **Contacts** | **Voltage** | **PW** | **Freq** |  |  |
| C1 | 0-3+ | 6.2 | 180 | 130 | 0-3+ | 6.4 | 180 | 130 | 12 | IC/BST |
| C2 | 0-1-2-c+ | 4.2 | 180 | 130 | 0-1-2-c+ | 4.3 | 150 | 130 | 6 | ITP |
| C3 | 1-2-c+ | 5.4 | 300 | 130 | 1-2-c+ | 5.4 | 300 | 130 | 10 | IC/BST |
| C4 | 1-c+ | 3.6 | 210 | 130 | 1-c+ | 3.6 | 210 | 130 | 15 | IC/BST |
| C5 | 1-3+ | 9 | 330 | 130 | 1-3+ | 9 | 330 | 130 | 10 | IC/BST |
| C6 | 0-1+ | 4.2 | 270 | 130 | 0-1+ | 4.2 | 270 | 130 | 5 | IC/BST |
| C7 | 1-c+ | 8.0 | 60 | 100 | 1-c+ | 8.0 | 60 | 100 | 16 | IC/BST |

**Table S2: Stimulation parameters.** Parameters selected for crossovers after optimization period and at last follow-up. HAM-D scores at time of parameter selection.

IC/BST: internal capsule/bed nucleus of stria terminalis; ITP: inferior thalamic peduncle; PW: pulse width; Freq: frequency; HAM-D: Hamilton Depression Rating Scale (17-items); C1-7: patient 1-7

| **Test** | | **Baseline**  **(n = 7)**  **Mean (SD)** | **Crossover 2 OFF (n = 5)**  **Mean (SD)** | **Crossover 2 IC/BST (n = 5)**  **Mean (SD)** | **Crossover 2**  **ITP (n = 5)**  **Mean (SD)** |
| --- | --- | --- | --- | --- | --- |
|  |  |  |  |  |  |
| RAVLT | First trial list A | 4.9 (1.3) | 6.8 (2.6) | 7.8 (1.6) | 7.8 (4.1) |
|  | Total 5 trials list A | 42.4 (9.4) | 49.8 (12.9) | 51.6 (12.1) | 48.6 (16.7) |
|  | Interference list B | 3.9 (1.3) | 4.0 (0.7) | 5.0 (1.2) | 4.4 (0.9) |
|  | List A immediate recall | 8.9 (2.8) | 10.0 (2.7) | 11.6 (3.2) | 10.8 (2.7) |
|  | List A late recall | 9.3 (2.6) | 10.6 (2.5) | 11.8 (3.3) | 11.4 (2.5) |
| TMT | A | 37.6 (10.2) | 34.4 (11.0) | 28.2 (8.1) | 31.2 (12.5) |
|  | B | 97.0 (60.3) | 81.2 (25.9) | 68.4 (21.2) | 70.0 (14.0) |
| Stroop | Color | 65.3 (13.7) | 64.0 (17.9) | 52.4 (9.0) | 59.2 (11.9) |
|  | Color-word | 120.9 (29.4) | 108.0 (33.0) | 93.2 (28.7) | 102.0 (28.3) |
| ROCFT | Copy | 33.0 (2.9) | 33.6 (2.8) | 34.8 (2.2) | 33.6 (2.9) |
|  | Immediate recall | 15.3 (6.2) | 17.8 (5.1) | 21.0 (5.7) | 18.4 (6.0) |
|  | Late recall | 16.4 (5.9) | 19.6 (4.4) | 22.6 (5.9) | 20.6 (6.9) |
| DSpT | Max span forward | 5.1 (0.9) | 5.4 (0.5) | 5.8 (0.4) | 5.8 (0.4) |
|  | Max span back | 4.6 (0.5) | 4.8 (0.4) | 4.6 (0.5) | 5.2 (0.4) |
| WFT | Total words (3 letters) | 31.7 (13.5) | 34.6 (14.4) | 37.8 (13.9) | 32.6 (12.0) |
| SPM of Raven | | 38.7 (11.5) | 40.7 (10.8) | 40.8 (13.1) | 44.0 (11.0) |

**Table S3: Neuropsychological evaluation**. Descriptive statistics (mean and standard deviation) for neuropsychological test scores during the second crossover.

OFF: no stimulation; IC/BST : internal capsule/bed nucleus of stria terminalis stimulation; ITP: inferior thalamic peduncle stimulation; RAVLT: Rey Auditory Verbal Learning Test; TMT A & B: Trail Making Test A & B; Stroop: Stroop Test; ROCFT: Rey-Osterrieth Complex Figure Test; SPM of Raven: Standard Progressive Matrices of Raven; DSpT: Digit Span Test; WFT: Word Fluency Test

| **Scale** | **Baseline** | **3YFU** | **LFU** |
| --- | --- | --- | --- |
| MADRS | 38.1 (7.0) | 13.0 (7.4) | 15.0 (5.7) |
| IDS | 48.4 (10.5) | 22.3 (11.4) | 22.1 (7.4) |
| BHS | 16.4 (3.2) | 9.1 (5.8) | 8.6 (3.7) |
| HAM-A | 24.1 (9.0) | 8.0 (4.1) | 11.1 (6.4) |
| YMRS | 0.7 (1.3) |  | 1.7 (1.7) |
| CGI-S | 5.6 (0.5) | 2.0 (1.0) | 1.9 (0.9) |
| CGI-I |  | 5.0 (1.2) | 5.0 (1.2) |
| PGI-S | 5.6 (0.5) | 1.9 (1.6) | 2.1 (0.9) |
| PGI-I |  | 4.9 (1.2) | (4.9) 1.1 |
| GAF | 41.4 (3.8) | 66.4 (10.7) | 64.3 (6.7) |

**Table S4:** **Secondary outcome measures during follow-up.** Secondary outcome measures at baseline, at 3 year follow-up (3YFU) and at last follow-up (LFU). Note that for patients C6 and C7, LFU coincided with 3YFU, while LFU data were collected later in the other patients.

MADRS: Montgomery-Åsberg Depression Rating Scale; IDS: Inventory for Depressive Symptoms; BHS: Beck Hopelessness Scale; HAM-A: Hamilton Anxiety Rating Scale; YMRS: Young Mania Rating Scale; CGI-S: Clinical Global Impressions – Severity; PGI-S: Patient Global Impressions – Severity; GAF: Global Assessment of Function

| **Adverse Events – Surgery/Device Related** | | | |
| --- | --- | --- | --- |
| **Adverse Event** | | | **#** |
| conversely labelled leads (IC/BST leads labelled as ITP leads and vice versa)* | | | 1 |
| damage of IC electrode* | | | 1 |
| infection around IPG site* | | | 2 |
| infection of stereotactic frame attachment site | | | 1 |
| triggering of theft alarm | | | 1 |
| uncomfortable sensation around IPG | | | 1 |
| **Adverse Events – Stimulation Induced** | | | |
| **IC/BST** | **#** | **ITP** | **#** |
| **Psychiatric** | | | |
| agitation | 2 | anxiety | 2 |
| anxiety | 3 | apathy | 1 |
| disruption of social skills | 1 | compulsive stealing | 1 |
| feeling of derealization | 1 | hyperventilation | 1 |
| hearing voice of deceased sister | 1 | irritability | 1 |
| hyperventilation | 1 | mood swings | 1 |
| increase in impulsivity | 1 | nervosity | 1 |
| intrusive thoughts about violent suicide | 1 | obsessive counting | 1 |
| mood swings | 1 | sleep disturbances | 4 |
| nervosity | 4 | tiredness | 2 |
| panic attack | 3 |  | |
| sleep disturbances | 6 |  |  |
| tiredness | 4 |  |  |
| **Neurological/neurocognitive** | | | |
| concentration difficulties | 2 | extrapyramidal symptoms | 1 |
| disturbed balance | 2 | nystagmus | 1 |
| dizziness | 1 | transient confusion | 1 |
| feeling of twitches in neck and face | 1 |  | |
| headache | 1 |  |  |
| memory complaints | 5 |  |  |
| transient confusion | 2 |  |  |
| transient paresthesia left side of face | 1 |  |  |
| **Urinary/Sexual** | | | |
| delayed ejaculation | 1 | difficulty voiding bladder | 1 |
| difficulty voiding bladder | 1 | libido decrease | 1 |
| erectile dysfunction | 1 | libido increase | 1 |
| libido decrease | 2 |  | |
| transient urinary incontinence | 1 |  |  |
| urinary retention | 1 |  |  |

| **Other** | | | |
| --- | --- | --- | --- |
| cough | 1 | nausea | 1 |
| family problems due to increased assertiveness | 1 | sleep apnea | 1 |
| feeling of warmth | 1 | sweating | 1 |
| irregular bowl movement | 1 | urge to empty bowels | 1 |
| locked jaw | 1 | weight gain | 1 |
| weight gain | 1 |  | |
| **Adverse Events – Not Related to DBS** | | | |
| **IC/BST** | **#** | **ITP** | **#** |
| **Disorder Related** | | | |
| abuse of alcohol | 2 | increase in depressive symptoms | 7 |
| abuse of benzodiazepines | 1 | increase of suicidal thoughts | 2 |
| deliberate self-harm* | 1 |  | |
| increase in depressive symptoms | 7 |  |  |
| increase of suicidal thoughts | 4 |  |  |
| suicide* | 2 |  |  |
| suicide attempt* | 1 |  |  |
| **Other** | | | |
| appendicitis* | 1 | back pain | 1 |
| fall from stairs* | 1 | cold | 1 |
| fall on ice, humerus fracture* | 1 | fall from bike | 1 |
| family problems | 4 | family problems | 1 |
| flu | 1 | meniscus tear | 1 |
| hypokalemia | 1 |  | |
| inguinal hernia* | 1 |  |  |
| otitis externa | 1 |  |  |
| rhinitis | 1 |  |  |
| sternal pain | 1 |  |  |
| stomach pain | 1 |  |  |
| zona | 1 |  |  |

**Table S5: Adverse Events.** Adverse events (AEs) reported during the course of this trial. AEs are ordered per category and alphabetically. It sufficed that the patient complained once about something to list this in the AEs. AEs are reported separately for IC/BST stimulation (341 follow-up months) and ITP stimulation (98 follow-up months). Psychiatric symptoms (e.g., sleep disturbances) that were reported by the patient without an increase in other depressive symptoms are mentioned separately. When the patient reported an increase in several depressive symptoms, ‘increase in depressive symptoms’ was registered.

IC/BST: internal capsule/bed nucleus of stria terminalis stimulation; IPG: implanted pulse generator; ITP: inferior thalamic peduncle stimulation; #: number of patients reporting adverse event; *: serious adverse events
